# Sex Differences in Albumin Quotient and Cerebrospinal Fluid Total Protein Content Do Not Depend on Anthropometric Factors

**DOI:** 10.3390/jpm14040362

**Published:** 2024-03-29

**Authors:** Massimiliano Castellazzi, Raffaella Candeloro, Caterina Trevisan, Samantha Permunian, Gaia Buscemi, Sara Ghisellini, Giovanna Negri, Giada Gilli, Caterina Ferri, Tiziana Bellini, Stefano Pizzicotti, Maura Pugliatti

**Affiliations:** 1 Department of Neurosciences and Rehabilitation, University of Ferrara, 44121 Ferrara, Italy; raffaella.candeloro@unife.it (R.C.); samantha.permunian@edu.unife.it (S.P.); gaia.buscemi@edu.unife.it (G.B.); giada.gilli2@studio.unibo.it (G.G.); tiziana.bellini@unife.it (T.B.); maura.pugliatti@unife.it (M.P.); 2 University Strategic Center for Studies on Gender Medicine, University of Ferrara, 44121 Ferrara, Italy; 3 Department of Medical Sciences, University of Ferrara, 44121 Ferrara, Italy; caterina.trevisan@unife.it; 4 Chemical-Clinical Analysis Laboratory, “S. Anna” University Hospital, 44124 Ferrara, Italy; s.ghisellini@ospfe.it (S.G.); g.negri@ospfe.it (G.N.); s.pizzicotti@ospfe.it (S.P.); 5 Department of Neuroscience, “S. Anna” University Hospital, 44124 Ferrara, Italy; caterina.ferri@unife.it

**Keywords:** cerebrospinal fluid, blood–cerebrospinal fluid barrier, albumin quotient, sex differences, anthropometric characteristics, sex-disaggregated analysis

## Abstract

(1) Background: Cerebrospinal fluid (CSF)/serum albumin quotient (QAlb) and CSF total protein (TP) are more elevated in males than females, and this has been hypothesised to be due to anthropometric differences between the sexes. This study aimed to investigate QAlb and CSF TP as a function of body height, weight, and body mass index (BMI). (2) Methods: A total of 207 patients were included in the study and analysed blinded to clinical diagnosis. (3) Results: Multivariable linear regressions were run to predict log-transformed Qalb and log-transformed CSF TP value from age, sex, weight, and height (first model) or from age, sex, and BMI (second model). In both models, age (β = 0.004, 95% CI = 0.002 to 0.006) and sex (β = −0.095, 95% CI = −0.169 to −0.021, and β = −0.135, 95% CI = −0.191 to −0.079) were significant predictors for QAlb, but weight, height, and BMI were not. Similarly, age (β = 0.004, 95% CI = 0.003 to 0.006) and sex (β = −0.077, 95% CI = −0.142 to −0.013, and β = −0.109, 95% CI = −0.157 to −0.060) were significant predictors for CSF TP, while anthropometric characteristics were not. No differences in QAlb and CSF TP were found when grouping males and females by BMI status. (4) Conclusions: Our data suggest that anthropometric characteristics could not explain the sex-related differences in QAlb and CSF TP.

## 1. Introduction

Brain sexual dimorphisms notably impact brain anatomy, biochemistry, and various psychological and cognitive processes [1,2].

Men and women present pronounced differences in the susceptibility, onset, progression, and severity of neurological and psychiatric diseases [2]. Men are more likely to be diagnosed with Parkinson’s disease [3], attention deficit hyperactivity disorder [4], and autism spectrum disorders [5] and are prone to present an early onset of schizophrenia spectrum disorders [6]. Women, however, are more likely to develop depression, anxiety [7], Alzheimer’s disease [8], and multiple sclerosis [9]. 

Studies on the differences in brain structure revealed that males, on average, have almost 10% larger brain volumes compared to females, and this sexual dimorphism is not diffusely distributed across the brain but rather is region-specific [10]. On the one hand, women have a higher percentage of grey matter, while on the other hand, men have a higher percentage of white matter, greater lateral ventricles, and higher sulcal and cerebrospinal fluid (CSF) volumes than women [2]. Moreover, CSF in men presents a higher density than in women [11].

CSF is a body fluid that permeates the central nervous system (CNS), whose composition is mainly like a plasma ultrafiltrate with a lower protein content and very few immune cells [12]. CSF is predominantly, but not exclusively, secreted by the choroid plexuses within the cerebral ventricles. Brain interstitial fluid, ependyma, and capillaries also contribute to CSF secretion [13]. Lumbar puncture, or spinal tap, is a mini-invasive procedure that allows the withdrawal of CSF from the spinal subarachnoid space. To date, CSF analysis and brain biopsy are the only tools to investigate the presence of inflammation within the CNS [14].

Two crucial roles are attributed to CSF: (i) a mechanical function, where it serves to shield the brain and spinal cord from mechanical stresses arising from movements and the positioning of the head in space; (ii) a biological function, as CSF serves as the guardian of cerebral homeostasis, maintaining stable physicochemical characteristics under normal conditions when these may be disrupted in various central nervous system (CNS) and peripheral nervous system (PNS) pathologies [13].

The CNS is shielded from harmful substances within the bloodstream primarily by two structures: (i) the blood–brain barrier (BBB), situated between the vascular system and the brain tissue, and (ii) the blood–CSF barrier located at the choroid plexuses within the cerebral ventricles [12].

Choroid plexuses are complex structures consisting of epithelial and endothelial layers encompassing a highly vascularised core surrounded by connective tissue and tightly bound epithelial cells [15].

The blood–CSF barrier, composed of various anatomical components, actively manages the passage and filtration of large molecules from the blood into the CSF. The protein content in CSF is governed and upheld by the integrity of the blood–CSF barrier alongside the CSF flow rate [16]. In neonates, both pre-term and full-term, CSF protein concentrations are initially elevated but tend to decrease gradually during the first year of life, persisting at low concentrations throughout childhood. Conversely, in adults, CSF protein concentrations tend to increase with age [17]. The albumin quotient (QAlb), calculated as the ratio of albumin concentration in CSF to that in serum, serves as an indicator of the blood–CSF barrier’s integrity [14]. QAlb remains unaffected by intrathecal protein synthesis since CSF albumin is entirely derived from plasma, thus forming an essential component of mathematical formulas for quantifying intrathecal immunoglobulin synthesis. QAlb is a standardised value unaffected by methodological variations, as CSF and serum albumin concentrations are typically measured using nephelometry or turbidimetry, ensuring consistent reference values across different laboratories [14].

CSF protein concentration and QAlb exhibit a gradient along the CSF circulation within the neuroaxis, with lower concentrations observed at the ventricular level and higher concentrations in the lumbar sac, forming what is commonly referred to as a “rostral-caudal protein gradient” [16].

An analysis of the CSF should involve, among other steps, (i) determination of the total protein (TP) and cellular content, (ii) evaluation of the integrity of the blood–CSF barrier through calculation of the QAlb, and (iii) investigation of the presence of intrathecal synthesis of antibodies [14]. Among these laboratory parameters, recent studies have highlighted that men are more likely to present blood–CSF barrier dysfunction than women, with higher QAlb values and a consequent increase in TP content in the CSF, regardless of age and pathology. In particular, sex-related differences in blood–CSF barrier permeability and CSF protein content have been observed in patients with multiple sclerosis and other inflammatory and non-inflammatory neurological disorders [18], as well as in psychiatric patients [19] and individuals with neurodegenerative conditions [20]. These differences have also been confirmed in subjects without a specific diagnosis of neurological disorder [18,21,22,23,24,25] and in healthy controls [24].

Greater body height in males, resulting in a higher rostro-caudal gradient, has been hypothesised as one potential cause of these sex-related differences [19]. Furthermore, barrier function appeared to be influenced by other disease-independent determinants, such as weight and body mass index (BMI) [26].

The present study aimed to investigate, for the first time, whether the QAlb and CSF TP content differ as a function of anthropometric characteristics such as body height, weight, and BMI in patients with neurological diseases, with the goal of exploring potential corrective measures to address sex-related disparities.

## 2. Materials and Methods

### 2.1. Study Design

This was a retrospective analysis that included patients admitted to the S. Anna University Hospital of Ferrara (northern Italy) who underwent diagnostic lumbar puncture for suspected neurological disease. The study was approved by the Medical Ethics Committee in Research “Comitato Etico di Area Vasta Emilia Centro della Regione Emilia-Romagna” (protocol number 770/2018/Oss/AOUFe).

Clinical and laboratory data were collected anonymously. Both pre-analytical and analytical procedures were performed according to good clinical practice and following international guidelines [14]. The study did not include additional procedures for patients and the healthcare system. Written informed consent was obtained from each patient upon admission. For all enrolled subjects, the “time of the study” refers to the moment they underwent lumbar puncture for diagnostic purposes.

### 2.2. Sample Size Calculation

The minimum size was calculated through ClinCalc.com, considering a study population constituted by two independent groups: (i) male and (ii) female patients. The primary endpoint was the blood–CSF barrier dysfunction, determined as Qalb values greater than the threshold “age/15 + 4” (in our population: 44.4% of males vs. 17.4% of females), and a female/male ratio of 2.3 was also considered. The calculation returned a minimum sample size of 102 subjects, including 31 males and 71 females.

### 2.3. Study Population

The study population was composed of 207 consecutive patients (63 males and 144 females), hospitalised at the Neurology Units of Ferrara. As in previous studies, all analyses were performed blinded to clinical suspicion and definitive diagnosis [23,24,27].

For each patient, body height and weight data were collected during hospitalisation, and BMI was calculated with the formula: BMI = weight(kg) ÷ height(meters)^2^. Males and females were further grouped on their BMI weight status categories as follows: (i) underweight, if BMI was less than 18.5 (0 males, 12 females); (ii) healthy weight, if BMI was in the range of 18.5 to 24.9 (28 males, 94 females); (iii) overweight, if BMI was between 25.0 and 29.9 (31 males, 34 females); (iv) obese, when BMI was greater than 30.0 (9 males, 17 females).

Exclusion criteria were CSF white blood cells > 10/μL, the presence of CSF discolourations, and CSF repeated samples [23].

### 2.4. Cerebrospinal Fluid Analysis

Paired blood and CSF samples were obtained from each patient at the same time as a part of the diagnostic work-up. Cell-free CSF and serum were obtained after centrifugation at 2000× *g* at room temperature. Samples were analysed immediately after centrifugation or stored in aliquots at −80 °C until examination. All analyses were performed on the 2nd–4th mL of CSF. For each patient, albumin concentrations were measured in CSF and serum samples by immunochemical nephelometry with the Beckman Array Protein System or the IMMAGE 800 immunochemical system (Beckman Instruments, Fullerton, CA, USA) according to the procedure described by Salden [28]. For each subject, serum and CSF samples were tested in the same run, and albumin concentrations were expressed as mg/L (CSF) and g/L (serum). The blood–CSF barrier function was evaluated with the use of Qalb according to the formula: Qalb = [albumin]CSF ÷ [albumin]serum.

In accordance with the literature, two different age-dependent formulas were utilised to calculate the upper reference limit (URL) of Qalb for each subject included in the study. Specifically, the formula URL = age/15 + 4 [29] was employed in routine clinical practice, while the second formula URL = age/25 + 8 [17] was utilised solely for research purposes during the drafting of this manuscript.

CSF TP concentrations were measured with the Beckman Coulter AU640/AU640e automated chemistry analyser, as published before [23]. In routine analysis, the URL of 500 mg/L was used for each subject. In a second analysis, the 500 mg/L URL was applied only to patients younger than 30 years, while an age-adjusted URL of 600 mg/L was applied to patients as young as or above 30 years [17].

### 2.5. Statistical Analysis

After checking normality by evaluating the graphical distribution of the variables and using the Kolmogorov–Smirnov test, normally distributed quantitative variables were presented as the mean and standard deviation (SD), while non-normally distributed data were expressed as the median and interquartile range (IQR). The comparisons between males and females were carried out using the Student’s *t*-test and the Mann–Whitney test, as appropriate. Categorical variables were reported as counts (percentages), and Fisher’s exact test was used to compare them between groups. Correlations between QAlb and CSF TP concentrations with anthropometric measures (body height, weight, and BMI) were performed using the non-parametric Spearman test.

The association of QAlb and CSF TP with the demographic (age, sex) and anthropometric variables was evaluated through multivariable linear regression. For this model, log-transformed QAld and CSF TP variables were considered as dependent variables, while age, sex, weight, height, and BMI were considered as independent variables. The strength of these associations was expressed as β coefficients and the 95% confidence interval (95% CI).

A Kruskall–Wallis test with Dunn’s post hoc test was used to perform multiple comparisons of QAlb and CSF TP concentrations between patients with different BMIs.

Two-tailed *p*-values less than 0.05 were considered statistically significant for all statistical analyses. Prism 10 software for MacOS (GraphPad Software, La Jolla, CA, USA) and the IBM Statistical Package for the Social Sciences (SPSS) (IBM Corp., Armonk, NY, USA) version 21.0 for Windows were used to perform the statistical analyses.

## 3. Results

### 3.1. Patient Characteristics

The demographic, anthropometric, and laboratory data of the 207 patients included in the study are shown in Table 1.

As reported, compared to females, males were more likely to be older and to have a higher weight, height, and BMI. Considering laboratory parameters, males had higher CSF albumin concentrations (284.1 vs. 184.1 mg/L, *p* < 0.0001), QAlb values (6.6 vs. 4.61, *p* < 0.0001), and CSF TP concentrations (500.0 vs. 360.0 mg/L, *p* < 0.0001) than females, while no differences were found for serum albumin (Table 1). Moreover, males had a higher prevalence of (i) brain–CSF barrier dysfunction, as assessed by QAlb values exceeding age-adjusted thresholds, and (ii) elevated CSF TP concentrations surpassing both age-adjusted and unadjusted thresholds (Table 1).

### 3.2. Correlations between Albumin Quotient, Cerebrospinal Fluid Total Protein Concentrations, and Anthropometric Characteristics

QAlb correlated positively with CSF TP in males (Spearman r = 0.720, *p* < 0.001) and females (Spearman r = 0.838, *p* < 0.001).

As reported in Table 2, QAlb positively correlated with weight (r = 0.243, *p* < 0.001), height (r = 0.243, *p* < 0.001), and BMI (r = 0.143, *p* = 0.04) across the entire cohort; however, these correlations did not emerge when disaggregating by sex.

Similar results were observed for CSF TP concentrations that positively correlated with weight (r = 0.232, *p* = 0.001) and height (r = 0.241, *p* = 0.001) in the total population but did not correlate with anthropometric characteristics in sex-disaggregated subgroups (Table 3).

### 3.3. Multiple Linear Regression Analysis

Table 4 shows the results of the linear regression for the association between sociodemographic and anthropometric variables and QAlb and CSF TP concentration.

When considering the QAlb, we found that either age, sex, weight, and height (Model 1), or age, sex, and BMI (Model 2) could explain around 23% of the outcome variability (R^2^ = 0.237 for Model 1, and R^2^ = 0.227 for Model 2). Age (β = 0.004, 95% CI = 0.002 to 0.006 in both models) and sex (β = −0.095, 95% CI = −0.169 to − 0.021, and β = −0.135, 95% CI = −0.191 to −0.079) were independently associated with albumin quotient in both statistical models, while non-significant results emerged for anthropometric factors (Table 4).

Regarding CSF TP values, the variables included in Models 1 and 2 explained about 25% of the outcome variance (R^2^ = 0.253 for Model 1, and R^2^ = 0.245 for Model 2). As above, no significant associations were found between weight, height, or BMI and CSF TP values, while a direct and indirect association, respectively, was found for age (β = 0.004, 95% CI = 0.003 to 0.006 in both models) and sex (β = −0.077, 95% CI = −0.142 to −0.013, and β = −0.109, 95% CI = −0.157 to −0.060) (Table 4).

### 3.4. Albumin Quotient and Cerebrospinal Fluid Total Protein Concentrations in Patients Grouped by BMI

**Figure 1:**
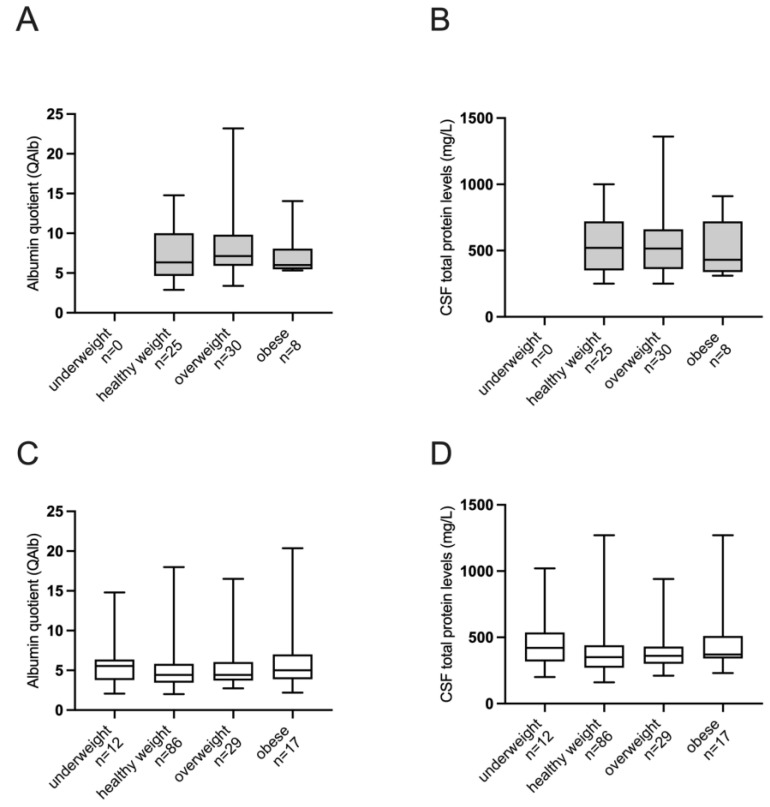
Albumin quotient (QAlb) (Panel **A** and **C**) and cerebrospinal fluid (CSF) total protein concentration distributions (Panel **B** and **D**) in males (grey) and females (white) subjects stratified by body mass index (BMI) weight status: underweight, BMI < 18.5; healthy weight, BMI in the range 18.5–24.9; overweight, BMI in the range 25.0–29.9; obese, BMI > 30.0. Kruskall–Wallis test followed by Dunn’s multiple comparisons test was used. No statistically significant differences were observed between groups.

As reported in Figure 1, no significant differences in QAlb and CSF TP concentrations were found when comparing BMI categories in males and females.

## 4. Discussion

This study suggests that anthropometric characteristics such as height, weight, and BMI cannot explain the sex differences in QAlb and CSF TP concentrations in patients undergoing lumbar puncture for diagnostic purposes.

As in previous studies, our population was analysed blinded to diagnostic suspicion [23,24,27]. According to general Italian population data [30], males had higher anthropometric measures than females. In line with the literature, they showed higher QAlb and CSF TP values than females [18,19,20,23,24,27], resulting in more frequent blood–CSF barrier dysfunction [18].

Despite the growing body of evidence on the existence of sexual dimorphism regarding QAlb and CSF TP concentrations, to date, the thresholds for these parameters are still applied regardless of sex [14,17].

The QAlb is widely accepted and used as the best tool to assess individual blood–CSF barrier function for blood-derived proteins in CSF [14]. This use is mainly because CSF albumin comes exclusively from the blood; therefore, its CSF/serum concentration quotient is representative of a diversity of sources and modes of diffusion through which serum proteins reach the CSF. Sex differences in body height, associated with a greater spine length and thus a longer distance for CSF flow in males than females, have been proposed to explain the gap between the sexes found regarding QAlb values [19]. However, in the present study, the independent association of QAlb and CSF TP values with sex remained significant even after adjusting for patients’ height.

Concerning weight and BMI, we found that these parameters correlated positively with QAlb and, partly, CSF TP, when analysing the total population, in accordance with a previous study [26]. However, such finding was not confirmed in the sex-stratified analyses and in the multivariable regressions where, as observed for body height, age, and sex, but not weight or BMI were independently associated with our outcomes. In other words, the sex differences in QAlb and CSF TP concentrations did not appear to be explained by height, weight, or BMI since the association between sex and the outcomes persisted even after including these anthropometric variables in the model. Overall, our study suggests the importance of stratifying the analyses by sex when evaluating QAlb and CSF TP concentrations and their relationship with anthropometric parameters. When the data are not disaggregated by sex, correlations may appear that disappear when the data are broken down. This happens because important variables like sex can influence both the independent and dependent variables. When the data are separated by sex, it becomes evident that the correlation might be due to sex differences within the sample, a phenomenon known as Simpson’s Paradox. This was well described by Baker and Kramer in their manuscript entitled “Good for women, good for men, bad for people. Simpson’s Paradox and the importance of sex-specific analysis in observational studies” [31].

Of note, we cannot exclude that our findings may be influenced by the distribution of BMI in the population. While our study includes Italian neurological patients, with a national obesity rate of 13%, a previous study was conducted in Germany [26], a population with an obesity rate of 21% [32]. In general, about half of Italians are overweight, while most Germans are overweight or obese [32]. This different distribution of body weight in populations could impact the results obtained in the respective analyses.

The lack of associations between QAlb and CSF TP with BMI in our population was also confirmed in a multi-comparison analysis in males and females grouped by BMI weight status. No differences in QAlb and CSF TP concentrations emerged when comparing underweight, normal weight, overweight, and obese subjects in the two sexes.

Taken together, our results are in line with a previous study that highlighted how sex differences in CSF density, of which CSF TP concentration is one of the main contributors, were not primarily driven by differences in body height and weight [11].

Ultimately, our data appear to confirm recent evidence about the importance of considering sex as an important variable that can explain, rather than confound, biomedical research findings [33].

Our research has no mechanistic ambitions and, due to its observational nature, we are therefore not able to conclude what causes the distribution of QAlb and CSF TP in the two sexes. Sex-related differences in blood–CSF barrier dysfunction could be secondary to multiple mechanisms, including the subarachnoid space size down the spine to the lumbar puncture site and/or the effect of sex hormones. Although our study seems to exclude the role of body length, an effect of a possible difference in the size of the subarachnoid space between males and females cannot be excluded from our data. Moreover, the female hormone 17β-estradiol [34] may influence the expression of enzymes involved in blood–CSF barrier breakdown [35,36], potentially leading to a protective effect [37]. Notably, despite stable QAlb sex-specific values during puberty or menopause, it is essential to consider the role of genetic predisposition associated with sex chromosomes, along with hormonal influences [2].

The present study has some limitations. The main one is the lack of a healthy population to include in this type of analysis. This is mainly due to the (mini)invasive nature of lumbar puncture, which is primarily performed for diagnostic purposes. Another limitation is the absence of clinical diagnosis. However, our primary objective was to study the independent impact of anthropometric traits on biochemical parameters of CSF analysis, including QAlb and CSF TP, regardless of the specific pathologies affecting individuals undergoing lumbar puncture. This research aimed to discern potential interventions to mitigate these influences, as has been implemented in the past, for example, by adjusting QAlb and CSF TP threshold values for age. However, the relationships between anthropometric characteristics and laboratory parameters did not emerge from our study. Furthermore, as we do not have anamnestic information on the enrolled subjects, we cannot even evaluate the effects of potential pre-existing conditions that could influence the efficiency of the blood–CSF barrier, such as vascular events, hypertension, and infectious or inflammatory diseases.

To reduce the presence of “contaminating factors”, as in previous studies, samples showing signs of blood contamination and excessive leukocyte content were excluded from the study [23,24,27].

## 5. Conclusions

Our research highlights that the gap between males and females in QAlb and CSF TP is not a consequence of anthropometric differences between the two sexes. In the era of personalised and gender-specific medicine, our results confirmed, therefore, the importance of conducting sex-disaggregated analyses.

Finally, while future studies are necessary to clarify the cause of this sex gap, on the other hand, new guidelines for CSF analysis that take patients’ sex into account in the applicability of QAlb and CSF TP thresholds are desirable.

## Figures and Tables

**Table 1:** Characteristics of the study population by sex.

	Males (*n* = 63)	Females (*n* = 144)	*p*
Age (years): mean ± SD	47.2 ± 17.4	40.4 ± 13.8	0.003
Weight (kg): median (IQR)	80.0 (69.0–90.0)	62.0 (54.0–70.8)	<0.0001
Height (m): median (IQR)	1.75 (1.72–1.81)	1.65 (1.59–1.69)	<0.0001
Body mass index: median (IQR)	26.3 (23.0–28.7)	22.7 (20.3–25.9)	<0.0001
CSF albumin (mg/L): median (IQR)	284.1 (210.6–390.4)	184.1 (146.5–251.5)	<0.0001
Serum albumin (g/L): mean ± SD	41.91 ± 5.06	41.03 ± 4.59	0.222
QAlb: median (IQR)	6.6 (5.4–9.7)	4.61 (3.6–6.1)	<0.0001
QAlb > age/15 + 4: n (%)	28 (44.4)	25 (17.4)	<0.0001
QAlb > age/25 + 8: n (%)	13 (20.6)	7 (4.9)	0.001
CSF TP (mg/L): median (IQR)	500.0 (360.0–710.0)	360.0 (290.0–447.5)	<0.0001
CSF TP > 500 mg/L: *n* (%)	32 (50.8)	23 (16.0)	<0.0001
CSF TP > 500 mg/L (age ≤ 30 years) and 600 mg/L (age ≥ 30 years): *n* (%)	23 (36.5)	13 (9.0)	<0.0001

Notes: Age and serum albumin concentrations had a normal distribution, and a Student *t*-test was used to compare males and females. All other variables showed a non-normal distribution, and the Mann–Whitney test was used to compare sexes. Categorical variables were compared with Fisher’s exact test. QAlb and CSF TP positivity was defined as a value above the reported upper reference limits. Abbreviations: CSF, cerebrospinal fluid; IQR, interquartile range; SD, standard deviation.

**Table 2:** Correlations of albumin quotient (QAlb) and anthropometric characteristics in the total sample and by sex.

	QAlb		
	vs. Weight	vs. Height	vs. BMI
All (*n* = 207)			
Spearman r	0.243	0.242	0.143
95% CI	0.111 to 0.367	0.110 to 0.368	0.007 to 0.274
*p*	<0.001	<0.001	0.040
Males (*n* = 63)			
Spearman r	0.126	0.174	0.0414
95% CI	−0.134 to 0.369	−0.085 to 0.410	−0.216 to 0.293
*p*	0.327	0.173	0.748
Females (*n* = 144)			
Spearman r	0.097	0.018	0.095
95% CI	−0.072 to 0.262	−0.151 to 0.186	−0.074 to 0.260
*p*	0.245	0.833	0.255

Abbreviations: BMI, body mass index; CI, confidence interval.

**Table 3:** Correlations of cerebrospinal fluid (CSF) total protein (TP) concentrations and anthropometric characteristics in patients disaggregated or not by sex.

	CSF TP		
	vs. Weight	vs. Height	vs. BMI
All (*n* = 207)			
Spearman r	0.232	0.241	0.133
95% CI	0.095 to 0.361	0.104 to 0.369	−0.007 to 0.269
*p*	<0.001	<0.001	0.056
Males (*n* = 63)			
Spearman r	0.095	0.133	0.009
95% CI	−0.164 to 0.341	−0.126 to 0.375	−0.248 to 0.263
*p*	0.460	0.299	0.947
Females (*n* = 144)			
Spearman r	0.052	0.002	0.050
95% CI	−0.117 to 0.219	−0.167 to 0.170	−0.120 to 0.216
*p*	0.533	0.984	0.556

Abbreviations: BMI, body mass index; CI, confidence interval.

**Table 4:** Multivariable regression analysis for the association between demographic and anthropometric variables with albumin quotient and cerebrospinal fluid total protein concentrations.

	Log Albumin Quotient			Log CSF Total Proteins		
	β	95% CI	*p*	β	95% CI	*p*
Model 1						
Age (years)	0.004	0.002, 0.006	<0.001	0.004	0.003, 0.006	<0.001
Female sex (ref: male)	−0.095	−0.169, −0.021	0.012	−0.077	−0.142, −0.013	0.019
Weight (kg)	0.001	−0.001, 0.003	0.386	0.000	−0.001, 0.002	0.707
Height (m)	0.228	−0.159, 0.615	0.247	0.204	−0.132, 0.541	0.233
Model 2						
Age (years)	0.004	0.002, 0.006	<0.001	0.004	0.003, 0.006	<0.001
Female sex (ref: male)	−0.135	−0.191, −0.079	<0.001	−0.109	−0.157, −0.060	<0.001
Body mass index	0.002	−0.04, 0.007	0.502	0.000	−0.004, 0.005	0.891

Abbreviations: CI, confidence interval; CSF, cerebrospinal fluid.

## Data Availability

The datasets used and analysed during the current study are available from the corresponding author upon reasonable request.
